# Mesalazine and Lactoferrin as Potential Adjuvant Therapy in Colorectal Cancer: Effects on Cell Viability and Wnt/β-Catenin Pathway

**DOI:** 10.3390/cimb47050327

**Published:** 2025-05-02

**Authors:** Joanna Słoka, Marcel Madej, Ilona Nowak, Barbara Strzałka-Mrozik

**Affiliations:** Department of Molecular Biology, Faculty of Pharmaceutical Sciences in Sosnowiec, Medical University of Silesia, 40-055 Katowice, Poland; joanna.sloka13@gmail.com (J.S.); mmarcel281297@gmail.com (M.M.); mc.ilona.nowak@gmail.com (I.N.)

**Keywords:** mesalazine, lactoferrin, colorectal cancer, iron metabolism

## Abstract

Colorectal cancer (CRC) remains one of the leading causes of cancer-related deaths, meaning it is essential to explore all possible strategies for its prevention and treatment. Unfortunately, risk factors such as an unhealthy lifestyle, lack of exercise, and obesity—which are increasingly prevalent in developed countries—contribute to CRC development. The aim of this study was to evaluate the effect of a mesalazine (MES) and lactoferrin (LACT) combination on the viability of CRC cells and healthy intestinal epithelial cells, as well as to assess the expression profile of target genes within the Wnt/β-catenin pathway. Additionally, this study aimed to preliminarily analyze the mechanism of action underlying the combined effects of these compounds. In this study, we used three CRC cell lines (HCT-116, DLD-1, and HT-29) along with the healthy intestinal epithelial cell line CCD 841 CoN. These cells were treated with MES and LACT separately, as well as in combination. We demonstrated that the combination of MES and LACT reduced the viability of CRC cells more effectively than either compound alone, while slightly increasing the viability of normal intestinal epithelial cells. The synergistic effect of MES and LACT may serve as a foundation for developing new treatment strategies for CRC, utilizing compounds with a high safety profile.

## 1. Introduction

Colorectal cancer (CRC) remains one of the greatest challenges in modern oncology, with approximately 1.9 million new cases diagnosed worldwide each year. Projections are concerning, as estimates suggest this number will surpass 3.2 million by 2040. The primary risk factors for CRC include unhealthy lifestyle habits such as smoking, excessive alcohol consumption, obesity, and physical inactivity [1].

While a genetic predisposition accounts for approximately 20% of cases, the majority of CRC cases result from complex interactions between environmental and epigenetic factors [2]. A key player in CRC pathogenesis is the Wnt/β-catenin signaling pathway, which, under physiological conditions, regulates embryogenesis and cellular homeostasis. However, dysregulation of this pathway—particularly its hyperactivation—drives tumor progression by promoting angiogenesis, epithelial–mesenchymal transition (EMT), and cancer cell proliferation. This dysregulation is associated with defects in the formation of the β-catenin degradation complex, which under normal conditions targets β-catenin for proteasomal degradation. When this complex is impaired, β-catenin accumulates in the cytoplasm, subsequently translocates into the nucleus, and binds to T-cell factor/lymphoid enhancer-binding factor (TCF/LEF) transcription factors, leading to the activation of target genes such as *CCND1* and *MYC*. Increased expression of these genes promotes excessive proliferation and contributes to tumorigenesis [3]. Additionally, aberrations in the Wnt/β-catenin pathway contribute to an increased population of cancer stem cells (CSCs), which are associated with chemoresistance and disease recurrence—two major challenges in effective CRC treatment [4,5,6].

The standard treatment for CRC includes surgical resection, chemotherapy—primarily regimens based on 5-Fluorouracil (5-FU)—and monoclonal antibodies targeting key molecular pathways [7]. Despite these aggressive treatment approaches, the efficacy of current therapies remains limited, highlighting the urgent need for novel therapeutic strategies to improve patient outcomes.

One promising research direction is drug repurposing, which involves utilizing compounds with well-established safety profiles for new therapeutic indications [8]. Within this framework, our study focuses on mesalazine (MES) (5-aminosalicylic acid, 5-ASA) and lactoferrin (LACT). MES, a nonsteroidal anti-inflammatory drug (NSAID), exhibits anticancer properties, making it a potential candidate for CRC prevention in patients with chronic intestinal inflammation [9,10,11]. Recent studies have highlighted MES’s ability to modulate key oncogenic pathways, including Wnt/β-catenin and NF-κB, thereby exerting antiproliferative effects in CRC models [12,13]. Similarly, LACT has attracted considerable attention for its ability to inhibit tumor growth and modulate immune responses, positioning it as a promising adjunct in anticancer therapies [14,15].

LACT is an iron-binding glycoprotein naturally present in milk and various human secretions, including saliva and tears [16]. It exhibits a broad spectrum of biological activities, including antibacterial, antiviral, antiparasitic, and anticancer properties [14]. Notably, as a naturally occurring molecule found in both the human body and food, LACT is considered entirely safe for therapeutic application [14,15].

**Figure 1:**
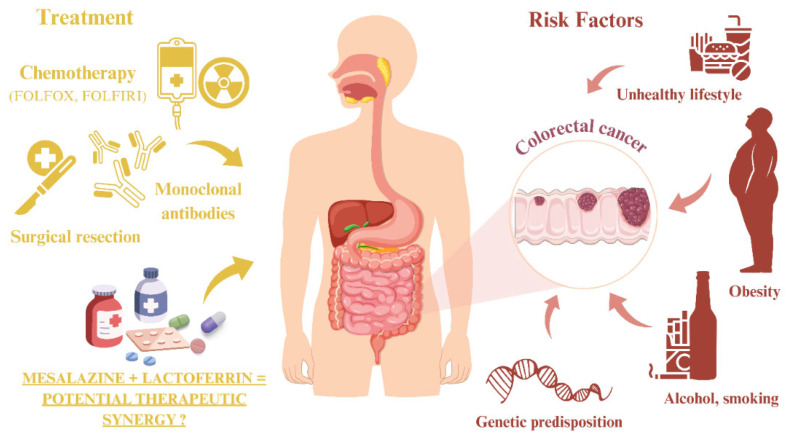
CRC treatment options and risk factors. The figure was partly generated using Servier Medical Art, provided by Servier, licensed under a Creative Commons Attribution 3.0 unported license.

Given the challenges in developing novel oncological therapies, our research strategy focuses on investigating the potential synergistic action of MES and LACT (Figure 1).

Given the increasing incidence and low 5-year survival rate of CRC patients [17,18], we conducted this study to evaluate the potential synergistic effects of MES and LACT on cancer cells. We analyzed the impact of MES, LACT, and their combination on CRC cell lines (HCT-116, DLD-1, and HT-29) as well as normal epithelial cells (CCD-841 CoN). Furthermore, we investigated the effects of these compounds on the expression of target genes within the Wnt/β-catenin pathway, a key regulator of CRC progression.

Our goal was to determine whether this combination synergistically inhibits cancer cell proliferation and could serve as a potential therapeutic approach for CRC treatment.

MES has been used for decades in CRC chemoprevention, while LACT demonstrates promising anticancer potential. Moreover, both compounds possess regenerative properties that support healthy tissue repair [19,20,21,22].

## 2. Materials and Methods

### 2.1. Cell Culture Conditions

Human CRC cell lines HCT-116 (CCL-247™, ATCC, Manassas, VA, USA), DLD-1 (CCL-221™, ATCC, Manassas, VA, USA), and HT-29 (HTB-38™, ATCC, Manassas, VA, USA), along with the normal intestinal epithelial cell line CCD 841 CoN (CRL-1790™, ATCC, Manassas, VA, USA), were incubated under standard conditions in culture flasks (Thermo Scientific, Waltham, MA, USA) at 37 °C in a 5% CO_2_ incubator (Direct Heat CO_2_; Thermo Scientific, Waltham, MA, USA).

Cells were cultured in different media depending on the cell line, respectively: HCT-116 in McCoy’s 5A medium (Sigma-Aldrich; Merck, St. Louis, MO, USA), DLD-1 in RPMI 1640 medium (Sigma-Aldrich; Merck, St. Louis, MO, USA), HT-29 in McCoy’s 5A medium (Sigma-Aldrich; Merck, MO, USA), and CCD 841 CoN in Eagle’s Minimum Essential Medium (EMEM) (Sigma-Aldrich; Merck, MO, USA). All media were supplemented with 10% fetal bovine serum (Euroclone S.p.A., Pero, Italy) and gentamicin (Sigma-Aldrich; Merck, MO, USA) at a concentration of 50 mg/L. Once cells reached 80% confluence, they were detached from the culture flasks using a standard trypsin-EDTA solution (Sigma-Aldrich; Merck, MO, USA) for further experimental procedures.

### 2.2. Compound Solutions and Cell Treatment

After reaching the appropriate confluence, cells were seeded into plates: 96-well plates (Thermo Scientific, Waltham, MA, USA) for cytotoxicity assays at a density of 10,000 cells per well, and 6-well plates (Thermo Scientific, Waltham, MA, USA) for molecular analysis at a density of 400,000 cells per well. Solutions were prepared by dissolving MES (item no. 70625; Cayman Chemical, Ann Arbor, MI, USA) and LACT (Sigma-Aldrich; Merck, MO, USA) in media specific to each cell line. The addition of NaOH was required for MES solutions to facilitate dissolution and maintain a pH of 7.0. All solutions were sterile-filtered using 0.2 µm disposable syringe filters (Sartorius, Göttingen, Germany) and protected from light throughout preparation.

After 48 h, the medium was replaced with MES solutions at concentrations of 10, 20, 30, 40, and 50 mM, LACT solutions at 100, 200, 400, and 800 µg/mL, and a combination of 30 mM MES with 100, 200, 400, and 800 µg/mL LACT. The control group consisted of cells incubated with the standard medium. For RT-qPCR analysis, cells in 6-well plates were treated with 30 mM MES, 400 µg/mL LACT, or a combination of 30 mM MES and 400 µg/mL LACT.

### 2.3. Cell Viability Assay

Cell viability was assessed using the MTT assay, following the methodology described in a previous study [23].

### 2.4. RT-qPCR

To establish the gene expression profile by RT-qPCR, cells from 6-well plates were lysed after 24 h of exposure to the test compounds using TRIzol (Invitrogen Life Technologies, Carlsbad, CA, USA). Total RNA was isolated according to the manufacturer’s instructions. The RT-qPCR procedure was previously described by Słoka et al. [23]. RT-qPCR reactions were performed using the Sensi-Fast™ reagent kit (Bioline, London, UK) with the following primer sequences: *MYC*: Forward, 5′-TCAAGAGGTGCCACGTCTCC-3′; Reverse, 5′-TCTTGGCAGCAGGATAGTCCTT-3′; *CCND1:* Forward, 5′-GAGCTGCTCCTGGTGAACAAG-3′; Reverse, 5′-GTGTTTGCGGATGATCTGTTTG-3′. Relative gene expression levels were calculated using the 2^−ΔΔCT^ method. The TATA-box binding protein (*TBP*) was selected as the reference gene for RT-qPCR normalization due to its stable expression across all experimental conditions and cell lines, as supported by Platet et al. [24] and our preliminary validation, in contrast to the variability observed with commonly used housekeeping genes such as *GAPDH* and *β-actin*.

### 2.5. In Silico Analysis

The ChemDIS-Mixture tool (version 2.4) (https://cwtung.nhri.edu.tw/chemdis/, accessed on 14 February 2025) was used to analyze proteins interacting with MES and LACT. This tool allowed us to estimate the potential effects of MES and LACT in combination by assessing their individual and joint interactions with target proteins.

Gene Ontology (GO) analysis was performed to evaluate the biological processes influenced by MES and LACT separately, as well as their combined effects. The analyses were based on hypergeometric tests, with multiple testing corrections applied using the Benjamini–Hochberg method and an adjusted *p*-value < 0.05 [25]. To prioritize potential interaction effects, we used a common *p*-value (Pjoint), where Pjoint represents the overall significance of the effects induced by MES and LACT simultaneously [26].

Molecular docking was performed for MES to analyze its interaction with selected target proteins: CCND1, MYC, and bovine LACT. The in silico study aimed to elucidate the mechanism of action and determine the strength and specificity of the binding between the ligand and these proteins.

The structure of MES was obtained from the PubChem database (https://pubchem.ncbi.nlm.nih.gov/, accessed on 16 February 2025) in the SDF format and subsequently converted to the mol2 format using ChimeraX 1.9 software. Crystal models of CCND1 (PDB ID: 2W69), MYC (PDB ID: 1NKP), and bovine LACT (PDB ID: 1BLF) were downloaded from the RCSB database (https://www.rcsb.org/, accessed on 18 February 2025) in the PDB format.

Prior to molecular docking, the protein structures were properly prepared. Using ChimeraX 1.9, the originally bound ligands, solvents, and other non-essential molecules were removed. Subsequently, using AutoDock Tools 1.5.7, water molecules were eliminated, hydrogen atoms were added, and electrostatic charges were calculated. The prepared structures were then saved in the PDBQT format.

Molecular docking was conducted using AutoDock Vina 1.1.2. To visualize the results, Discovery Studio 2025, ChimeraX 1.9, and LigPlot+ 2.2 software were employed.

### 2.6. Statistical Analysis

Statistical analysis was conducted using Statistica (version 13.3, Tibco Inc., Palo Alto, CA, USA). Each experimental condition was performed in triplicate. Qualitative data were visualized using box plots, while quantitative variables with asymmetric distributions were reported as the median (lower quartile–upper quartile).

The Shapiro–Wilk W test was used to assess the data distribution. Group comparisons were performed using the non-parametric Kruskal–Wallis ANOVA, followed by a multiple-comparisons test of mean ranks. A *p*-value < 0.05 was considered statistically significant.

## 3. Results

### 3.1. Viability Assessment Using the MTT Assay

**Figure 2:**
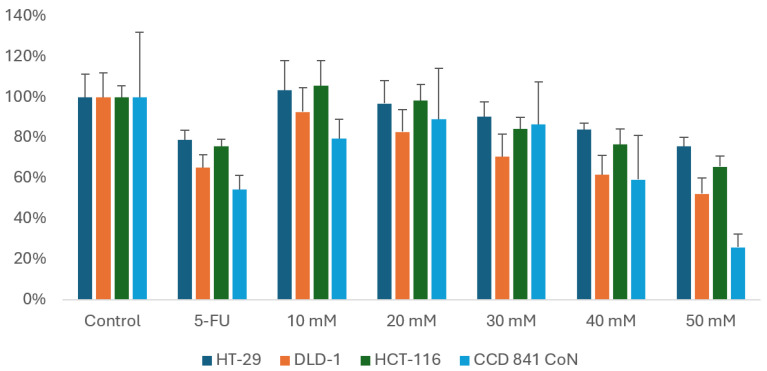
Changes in cell viability of CRC cell lines HT-29, DLD-1, and HCT-116, as well as the normal epithelial cell line CCD 841 CoN, after 24 h of exposure to MES, measured by the MTT assay. 5-FU was used as a positive control for cytotoxicity. Each experimental condition was performed in three biological replicates.

In our study, MES treatment resulted in a decrease in the viability of CRC cell lines DLD-1, HT-29, and HCT-116. This effect was dose-dependent, with higher MES concentrations leading to a more pronounced reduction in viability. A slight decrease in the viability of normal cells was also observed. The most significant reduction in cancer cell viability, while maintaining high viability of normal cells, was observed at 30 mM MES, which was selected for further experiments (Figure 2).

**Figure 3:**
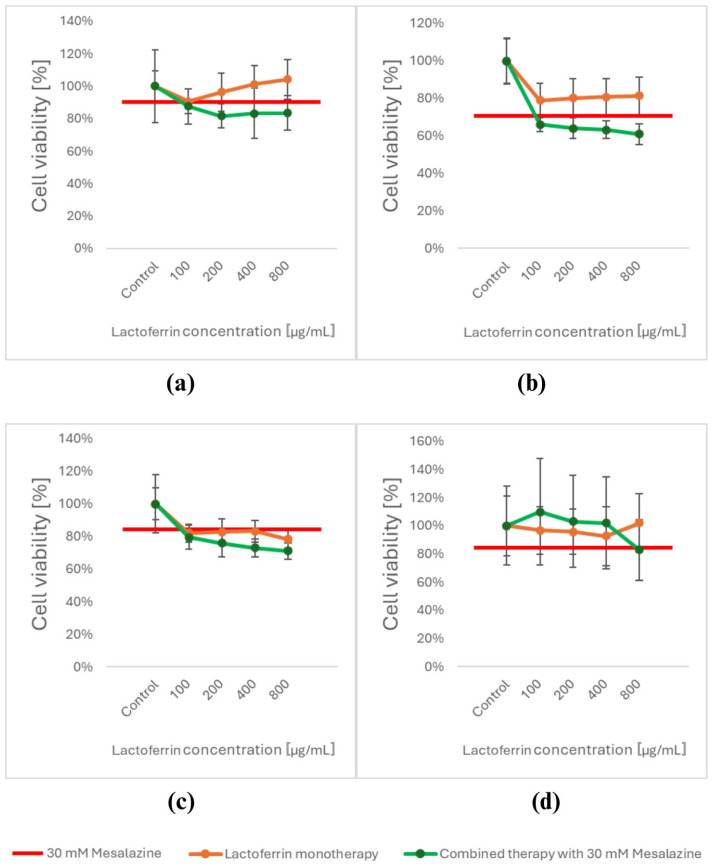
Changes in cell viability of CRC cell lines (**a**) HT-29, (**b**) DLD-1, and (**c**) HCT-116 and the normal epithelial cell line (**d**) CCD 841 CoN after 24 h of exposure to MES (30 mM), LACT (400 µg/mL), and their combination (MES with LACT), measured by the MTT assay. Data are presented as mean ± SD from three independent experiments. Although statistical analyses were performed, significance markers were not added to the figure to preserve clarity and avoid confusion regarding multiple comparisons.

After 24 h of exposure to LACT, a decrease in viability was observed in HT-29 cancer cells only at a concentration of 100 µg/mL (Figure 3a). In the DLD-1 cell line, viability decreased at all tested LACT concentrations, though the effect was less pronounced compared to MES monotherapy (Figure 3b). In HCT-116 cells, the reduction in viability following LACT exposure was comparable to that observed with MES monotherapy (Figure 3c). Normal cells treated with LACT showed no significant changes in cell viability (Figure 3d).

Due to the difficulty in determining the optimal LACT concentration, we decided to evaluate the cytotoxicity of the compound combination using MES at 30 mM with LACT at all tested concentrations (100, 200, 400, and 800 µg/mL).

Exposure to these combinations of MES and LACT resulted in a decrease in the viability of all CRC cell lines, with a significantly stronger effect compared to monotherapy with either compound. In contrast, an inverse correlation was observed in normal intestinal CCD 841 CoN cells, where treatment with MES and LACT at concentrations ranging from 100 to 400 µg/mL did not reduce viability but instead led to a slight increase in cell growth.

However, a significant decrease in normal cell viability was observed with the combination of 30 mM MES and 800 µg/mL LACT. Based on these findings, we selected 30 mM MES and 400 µg/mL LACT as the optimal combination for further studies.

### 3.2. Differential Expression of Wnt/β-Catenin Pathway Target Genes Based on Real-Time RT-qPCR

**Figure 4:**
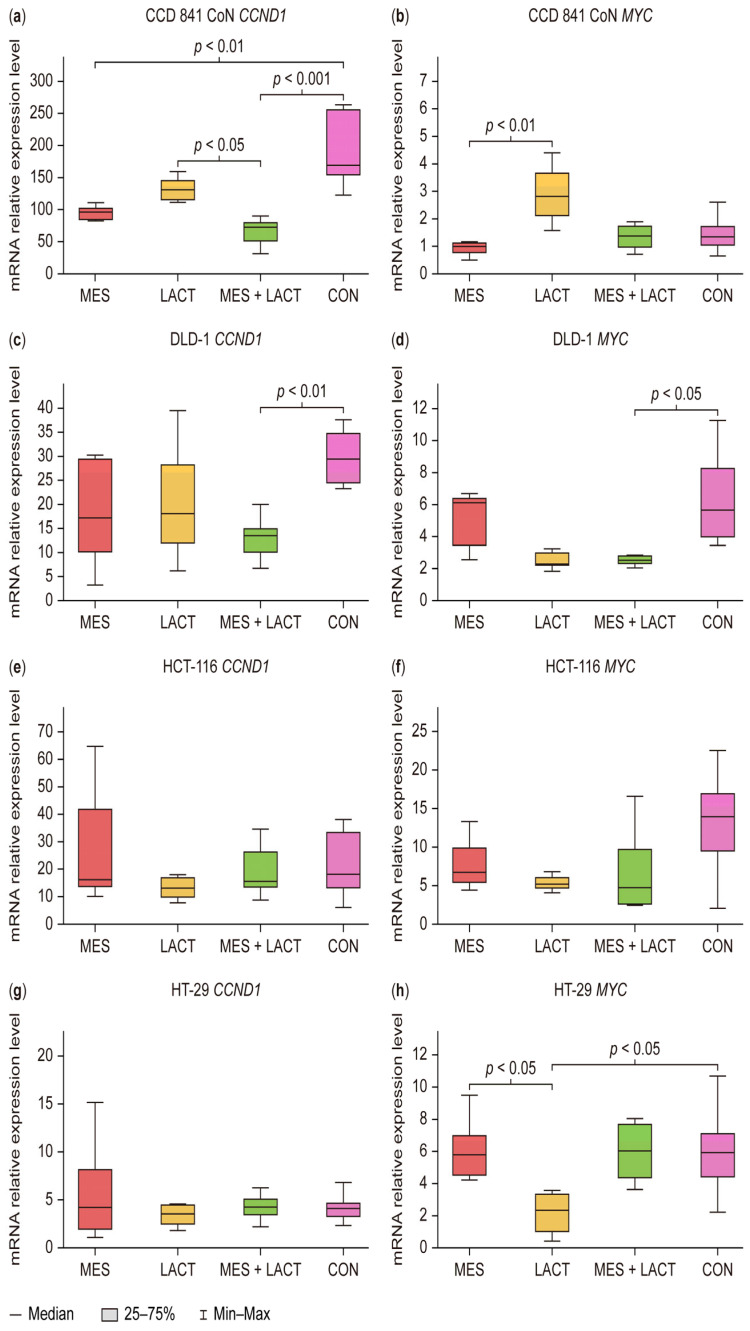
Changes in mRNA levels of (**a**) *CCND1* and (**b**) *MYC* in the CCD 841 CoN cell line, (**c**) *CCND1* and (**d**) *MYC* in the DLD-1 cell line, (**e**) *CCND1* and (**f**) *MYC* in the HCT-116 cell line, and (**g**) *CCND1* and (**h**) *MYC* in the HT-29 cell line after 24 h of treatment with 30 mM MES, 400 µg/mL LACT, 30 mM MES + 400 µg/mL LACT, and non-treated control cells (CON). Gene expression levels were normalized to the reference gene *TBP* and calculated using the 2^−ΔΔCT^ method. Box and whisker plots display the median, lower and upper quartiles, and minimum and maximum values. Abbreviations: MES—mesalazine; LACT—lactoferrin; CON—control (untreated).

The *MYC* and *CCND1* genes have been identified in the literature as target genes of the WNT/β-catenin signaling pathway [2]. Changes in their expression levels following treatment with the tested compounds are presented in Figure 4.

In the normal CCD 841 CoN colonocyte cell line, *CCND1* gene expression was significantly reduced following treatment with MES (*p* < 0.01) and the combination of MES and LACT (*p* < 0.001). Interestingly, LACT treatment also led to a decrease in *CCND1* expression, though this change was not statistically significant. Additionally, a significant difference was observed between cells treated with LACT and those with the combination of MES and LACT (*p* < 0.05) (Figure 4a).

For the *MYC* gene, a statistically significant difference in expression was noted between MES and LACT treatment (*p* < 0.05) (Figure 4b).

In the DLD-1 cell line, treatment with the combination of MES and LACT resulted in a statistically significant decrease in the expression of both *CCND1* (Figure 4c) and *MYC* (Figure 4d) (*p* < 0.05). Interestingly, LACT treatment also led to a reduction in *MYC* expression; however, this change was not statistically significant.

In the HCT-116 cell line, no statistically significant differences in the expression of the tested genes were observed between the treatment groups (Figure 4e,f).

In the HT-29 cell line, a variable expression pattern was observed for the *MYC* gene, while no statistically significant changes were detected in *CCND1* expression (Figure 4g). A statistically significant decrease in *MYC* expression was found following LACT treatment compared to the control (*p* < 0.05). Additionally, a significant difference was observed between MES and LACT treatments in this cell line (*p* < 0.05) (Figure 4h). Our findings are particularly important in light of the limited literature available on the impact of lactoferrin on the Wnt/β-catenin signaling pathway in cancer cells. Compared to MES, a well-known inhibitor of this pathway, LACT demonstrated a statistically significant reduction in *MYC* gene expression, suggesting its potential role in modulating oncogenic signaling. Additionally, the significant difference observed in viability reduction between MES and LACT treatments suggests that these agents may act through distinct mechanisms or differ in their potency. These results highlight the therapeutic value of combining MES and LACT to enhance anticancer efficacy in CRC models.

### 3.3. In Silico Prognosis of the Interaction of MES and LACT via ChemDIS-Mixture

In the next step, an in silico analysis was performed using the online tool ChemDIS-Mixture to predict the potential mechanism of action for the combination of MES and LACT. The analysis identified 184 proteins that may be affected by MES. Among these, two proteins were associated with anti-growth signaling (CDC25A, SRC), one with angiogenesis (PAK1), and two with metabolism (PTGS2, PTGS1). The proteins interacting with LACT include LF, SLC40A1, CSX, and OPRL1. The proteins with the highest scores are listed in Table 1.

Although no proteins were found to be directly affected by both MES and LACT simultaneously, bioinformatics analysis identified 14 shared target pathways influenced by both compounds (Table 2).

MES and LACT are associated with processes such as the negative regulation of apoptosis, maintenance of cellular redox homeostasis, iron ion binding, and positive regulation of protein phosphorylation.

### 3.4. Molecular Docking of MES

Molecular docking was performed for all proteins, namely bovine LACT, CCND1, and MYC, to determine the potential mechanism of action of MES. This technique enables the analysis of possible interactions with selected cellular proteins, providing an alternative to expensive and time-consuming experimental studies.

MES was classified based on its binding energy to the proteins (Table 3), with a lower ΔG value indicating stronger affinity. The most favorable parameters were obtained for LACT, whose binding energy was lower than those of CCND1 and MYC.

**Figure 5:**
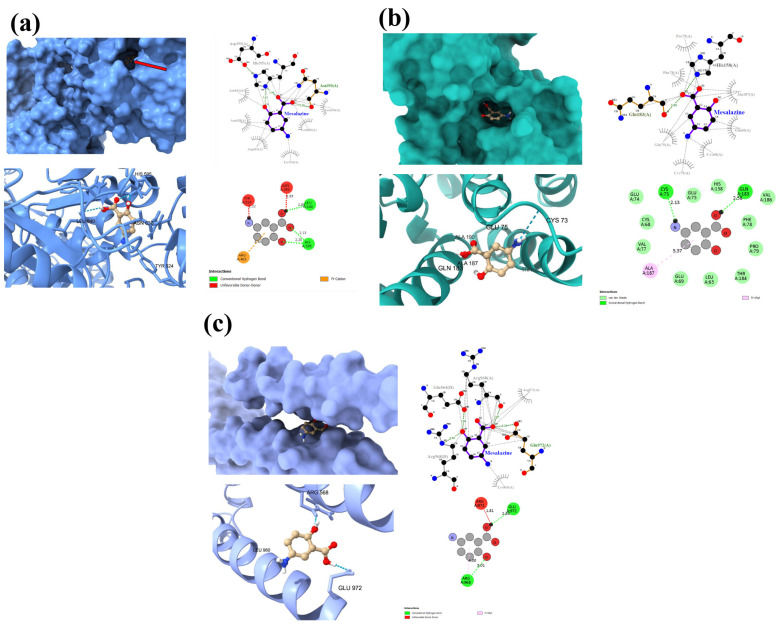
Graphical representation of MES interaction with LACT (**a**), CCND1 (**b**), and MYC (**c**). Red arrow (**a**) indicates a ligand (MES); green color indicates hydrogen bonds.

To further understand the interaction model, a binding analysis of the MES–protein complex was performed (Figure 5).

MES forms two hydrogen bonds with the Leu640 and His595 residues of bovine LACT (Figure 5a). In addition, the ligand exhibits numerous hydrophobic interactions with various protein residues. Despite its low binding energy, the complex also forms two unfavorable donor–donor bonds with Tyr524 and Asn393, which may affect its stability.

A similar pattern is observed in the MES-MYC complex (Figure 5c), where unfavorable donor–donor bonds are present alongside two hydrogen bonds with the Arg968 and Glu972 residues.

Interestingly, the MES-CCND1 interaction (Figure 5b), unlike the MES-LACT interaction, does not exhibit unfavorable donor–donor bonds. Moreover, MES forms two strong hydrogen bonds with the Gln183 and Cys73 residues of the CCND1 protein, as well as a pi-alkyl interaction and van der Waals interaction with Ala187.

## 4. Discussion

About one in ten cancer-related deaths is due to CRC. It is the second leading cause of cancer mortality, following breast cancer in women and lung cancer in men [27,28]. This underscores the urgent need for more effective and targeted therapeutic strategies for CRC treatment.

In line with the concept of drug repositioning, we aimed to explore a previously untested combination of existing compounds, with the objective that fundamental in vitro studies may have practical implications. The selected compounds, MES and LACT, have documented anticancer potential in the literature [11,13,29,30,31,32,33]. Additionally, we considered their favorable safety profile and tissue regenerative properties [19,21].

MES is a drug commonly used for the chemoprevention of CRC in patients with inflammatory bowel diseases, which predispose them to cancer development due to chronic inflammation [10]. Moreover, a study by Dixon et al. [11] demonstrated that MES exerts an inhibitory effect on CRC stem cells, which are often implicated in tumor recurrence.

The anticancer mechanisms of MES described in the literature primarily involve its inhibitory effects on the Wnt/β-catenin pathway. These effects include the inhibition of protein phosphatase 2A (PP2A) [34], blocking the β-catenin/TCF4 interaction, and sequestering β-catenin on the plasma membrane [35]. Additionally, MES has been shown to decrease PAK1 activity [36] and reduce the nuclear localization of β-catenin [37]. Moreover, MES influences the EGFR and PPARγ pathways, as well as the cell cycle, ultimately leading to tumor growth inhibition [38,39].

However, since PP2A also plays a critical role in regulating nuclear receptors such as the constitutive androstane receptor (CAR), its inhibition could potentially impact the expression of drug-metabolizing enzymes (DMEs), thereby affecting drug metabolism and safety [40]. Therefore, future studies should assess the expression profiles of key DMEs following MES and LACT co-treatment to exclude the possibility of adverse effects related to altered drug metabolism.

LACT is constitutively expressed in the human body, but an inverse correlation in gene expression for LACT and cancer incidence has been noted. This correlation was first observed in women with breast cancer in India. Furmanski et al. [41] observed lower levels of LACT-related RNAase activity in breast cancer patients.

An in vitro study conducted by Jiang and Lönnerdal [42] on the CRC cell line HT-29 demonstrated that bovine LACT reduces the viability of HT-29 cells, with apoptosis induction identified as the probable mechanism of action.

Kozu et al. [43] conducted a randomized, double-blind, controlled trial in patients with polyps predisposing them to cancer. Participants received either a placebo or LACT at doses of 1.5 g or 3.0 g daily for one year. Polyps were subsequently examined via colonoscopy. In patients who received the higher LACT dose (3.0 g), a reduction in colorectal polyp growth was observed. These studies indicate that higher doses of LACT are required to achieve an anticancer effect.

Mostafa et al. [22] further demonstrated that LACT has a beneficial role in mitigating the side effects of chemotherapy. After three months of oral LACT administration, patients experienced relief from mucositis, improved liver and kidney parameters, and an increase in red blood cell count. Additionally, the results confirmed that MES reduces the viability of cancer cells in the HT-29, DLD-1, and HCT-116 cell lines, while preserving a high viability of normal intestinal cells.

Given the anticancer potential of LACT, similar to MES, we explored a previously untested combination of these compounds. Notably, the combination of 30 mM MES and 400 µg/mL LACT resulted in a more pronounced reduction in cancer cell viability while enhancing the viability of normal colon cells. These findings suggest that the combination of MES and LACT may exhibit regenerative properties in normal intestinal epithelial cells while simultaneously exerting an inhibitory effect on cancer cells.

Overactivity of the Wnt/β-catenin pathway is implicated in the development, progression, metastasis, and recurrence of CRC [3]. A key regulator of this pathway is the β-catenin protein, specifically its level and localization. In the Wnt-off state, when Wnt ligands are absent, a degradation complex consisting of adenomatous polyposis coli (APC), casein kinase 1, glycogen synthase kinase 3 (GSK3β), and axin phosphorylates β-catenin, leading to its ubiquitination and rapid degradation in proteasomes. In the Wnt-on state, β-catenin is not ubiquitinated but instead accumulates in the cytoplasm and translocates to the nucleus. Once in the nucleus, it stimulates the expression of target genes, including *MYC* and *CCND1* [2].

In the DLD-1 cell line, only the combination of MES and LACT induced a statistically significant decrease in both *CCND1* and *MYC* expression, suggesting inhibition of the Wnt/β-catenin pathway. MES alone also inhibited the Wnt/β-catenin pathway, which is consistent with previous findings [36]; however, the effect was weaker compared to the combination treatment.

The HCT-116 cell line did not exhibit changes in *CCND1* and *MYC* gene expression in response to any of the tested compounds. There are limited data available for direct comparison with our findings. Only Bos et al. [36] demonstrated that MES inhibits the Wnt/β-catenin pathway in the DLD-1 cell line (β-catenin wild-type) but does not affect this pathway in HCT-116 cells (β-catenin mutant), which aligns with our results.

The genetic background of the CRC cell lines used in this study may partly explain the differential response to treatment. HCT-116 cells harbor a mutant β-catenin gene but wild-type *APC* and *p53*, whereas DLD-1 cells have wild-type β-catenin and mutations in both *APC* and *p53* genes [44]. These differences in key regulatory genes may influence the baseline activity of the Wnt/β-catenin and p53 pathways, thereby affecting the expression of downstream targets such as *CCND1* and *MYC*. Consequently, the distinct genetic profiles of these cell lines could contribute to the variability observed in response to MES and LACT treatment.

In the HT-29 cell line, LACT caused a decrease in *MYC* expression. However, in the viability assessment, a reduction in cell viability was observed after treatment with each compound separately, with a more pronounced effect following the combination treatment.

These findings suggest that the reduction in viability observed after MES, LACT, and their combination—except in the DLD-1 cell line—may not be directly related to Wnt/β-catenin pathway inhibition.

Bioinformatics analysis has shown that several proteins interacting with MES are strongly associated with tumor progression, including PAK1, TNF, SRC, and CDC25A.

p21-activated kinase 1 (*PAK1*) is expressed in both normal colon tissue and CRC, where it can participate in the regulation of other transcription pathways. *PAK1* is involved in the PI3K/AKT/mTOR pathway, contributing to tumor growth by activating β-catenin [45].

Tumor necrosis factor (TNF) is a cytokine closely linked to the tumor microenvironment, where it induces a persistent state of inflammation. Additionally, TNF is responsible for promoting the expression of angiogenic factors and enhancing tumor metastasis [46].

Src is a member of the membrane-associated non-receptor protein tyrosine kinase superfamily and is strongly associated with CRC, with overexpression reported in approximately 80% of patients. Increased *Src* expression is linked to metastasis and chemotherapy resistance [47].

In in silico analysis, four proteins (LF, SLC40A1, CSX1, OPRL1) interacted with LACT. Our study is in line with previous research in that after LACT treatment, the viability of cancer cells decreased, while the viability of normal intestinal epithelial cells increased. *SLC40a1* encodes ferroportin, which is responsible for the transport of iron out of the cell, thus closely monitoring cellular iron homeostasis. Iron management has a role in the tumorigenesis process; in some types of cancer such as breast, prostate, and lung cancer, *SLC40a1* expression is decreased [48].

In silico analysis identified two key processes involving both MES and LACT: iron ion binding and redox homeostasis, which may be interconnected. Although our bioinformatics analysis provides theoretical insights into the potential mechanisms of action of MES and LACT, further experimental studies—including protein-level validation, pathway activity assays, and in vivo functional analyses—are essential to confirm and fully elucidate the molecular mechanisms proposed by our in silico approach.

Iron metabolism plays a crucial role in maintaining the redox balance in many cancer cells, including CRC. Due to the increased activity of iron-dependent proteins involved in essential physiological processes such as cell cycle regulation, DNA synthesis, and angiogenesis, cancer cells have a heightened demand for iron [49]. At the same time, they are characterized by elevated levels of reactive oxygen species (ROS), resulting from their high metabolic activity and rapid proliferation. CRC is unique among malignancies in that it can acquire iron from two distinct sources: the intestinal lumen and the systemic circulation [50]. This increased iron requirement is associated with higher iron uptake and reduced iron efflux. Disruptions in iron homeostasis can be detrimental to cancer cells, meaning iron levels are tightly regulated by a network of proteins that control iron ion flow [51].

Iron metabolism is an attractive target for anticancer therapies, as both iron deficiency and excess can be damaging to cancer cells. Excess iron drives the Fenton reaction, generating large amounts of ROS, which disrupt redox homeostasis, induce lipid peroxidation, and ultimately lead to ferroptosis [52]. Iron also plays a regulatory role in the activation of several key signaling pathways that support cancer cell growth and survival. These include the Wnt/β-catenin pathway, which is particularly critical in CRC; the NF-κB pathway, associated with inflammation and cell survival; and the hypoxia-inducible factor 1-alpha (HIF-1α) pathway, which is stabilized under hypoxic conditions and elevated intracellular iron levels, promoting angiogenesis and a metabolic shift toward glycolysis [53].

MES may indirectly modulate oxidative stress pathways linked to iron metabolism, while LACT, as an iron-binding glycoprotein, can sequester free iron, reducing its bioavailability to cancer cells [54]. By limiting iron availability and enhancing oxidative stress, the combination of MES and LACT could impair tumor cell survival and proliferation. Furthermore, emerging evidence highlights that targeting iron metabolism can trigger ferroptosis—an iron-dependent form of regulated cell death—offering a promising strategy for CRC treatment [55,56]. These mechanisms may partially explain the enhanced anticancer effects observed in our study following MES and LACT co-treatment.

To identify a potential mechanism of action for MES on the CCND1, MYC, and LACT proteins, molecular docking was performed. This method facilitates the identification of potential interactions without the immediate need for additional experimental techniques, offering a valuable tool for preliminary screening. Such insights may contribute to the further optimization of active compounds, including the development of novel derivatives aimed at improving their biological efficacy [57,58].

Molecular docking of MES was also conducted by Li et al. [59]. In their study, an in silico analysis was performed to assess MES binding to the TNF, PTGS2, IL-1β, and EGFR proteins. The highest binding affinity for MES (−6.5 kcal/mol) was observed for the PTGS2 protein, which was consistent with our findings obtained using the ChemDIS-Mixture platform [59].

Moreover, our results demonstrate that MES forms hydrogen bonds with the studied proteins, particularly with CCND1, where no unfavorable donor–donor interactions were identified. This observation suggests the potential formation of a stable MES-CCND1 complex, which may contribute to the compound’s biological activity.

In the case of MYC, both favorable hydrogen bonds and unfavorable donor–donor interactions were observed, which may compromise the stability of the MES-MYC complex and suggest weaker interactions compared to CCND1. Conversely, the interaction between MES and LACT may stabilize the complex and enhance the immunomodulatory properties of LACT itself, potentially contributing to an increased anti-inflammatory effect in CRC.

Iron deficiency in cancer cells can lead to G1/S cell cycle arrest and apoptosis. Studies indicate that iron chelation can disrupt several signaling pathways, including AKT and Wnt, while also affecting autophagy, ultimately inhibiting tumor growth [60,61].

Further research is needed to determine the precise mechanism of action of the MES-LACT combination in cancer cells. However, it can be speculated that this effect is related to iron metabolism, given that LACT is an iron-transporting glycoprotein closely associated with ferroportin, which regulates iron efflux. Additionally, our previous studies have demonstrated that MES induces ferroptosis in DLD-1 cells [23].

To our knowledge, this is the first study to investigate the combined effect of MES and LACT on CRC cells and normal colonocytes. As such, there are no directly comparable data available in the current literature. While this novelty limits opportunities for direct comparisons, it underscores the significance of our findings as a foundation for future research. The distinct cellular responses we observed—strong inhibition of viability in CRC cells and a slight promotion of viability in normal colonocytes—likely result from fundamental biological differences between these cell types. Cancer cells typically exhibit an elevated iron demand, chronic oxidative stress, and hyperactivation of oncogenic signaling pathways, such as Wnt/β-catenin and NF-κB. These characteristics render them particularly vulnerable to therapeutic strategies targeting iron metabolism and the redox balance. In this context, LACT, through its iron-sequestering activity, and MES, through inhibition of β-catenin signaling and downstream effectors such as CCND1 and MYC, may act synergistically to disrupt cancer-specific vulnerabilities.

Although the exact mechanism of action of the MES-LACT combination remains unclear, our study provides a foundation for further research in this area. Both MES and LACT are considered safe for the human body, and their combined use demonstrates a beneficial effect on normal cells while inhibiting cancer cell proliferation—a highly desirable outcome in anticancer therapy.

## 5. Conclusions

Our study indicates that the combination of MES and LACT synergistically inhibits tumor cell growth while supporting the viability of normal intestinal epithelial cells. Gene expression analysis confirmed their role in modulating the Wnt/β-catenin pathway, particularly through the downregulation of *CCND1* and *MYC*, which are key in CRC progression.

Based on in silico analysis, the proposed anticancer mechanism may be linked to iron metabolism. However, further in vivo and clinical studies are needed to fully understand their therapeutic potential.

Given that MES is an FDA-approved drug and LACT is a naturally occurring, well-tolerated compound, our findings provide a strong basis for future research on this combination as a potential CRC therapy.

## Figures and Tables

**Table 1:** Proteins with the highest interaction scores for MES and LACT.

**MES**				
**Protein**	**Gene Symbol**	**Gene ID**	**Gene Name**	**Score**
**ENSP00000312304**	*TPMTD*	7172	thiopurine S-methyltransferase	0.981
**ENSP00000363512**	*ALOX5*	240	arachidonate 5-lipoxygenase	0.981
**ENSP00000287820**	*PPARG*	5468	peroxisome proliferator activated receptor gamma	0.968
**ENSP00000225275**	*MPO*	4353	myeloperoxidase	0.913
**ENSP00000388001**	*OAS1*	4938	2′-5′-oligoadenylate synthetase 1	0.864
**ENSP00000228928**	*OAS3*	4940	2′-5′-oligoadenylate synthetase 3	0.861
**ENSP00000342278**	*OAS2*	4939	2′-5′-oligoadenylate synthetase 2	0.861
**ENSP00000278568**	*PAK1*	5058	p21 (RAC1) activated kinase 1	0.820
**ENSP00000356438**	*PTGS2*	5743	prostaglandin-endoperoxide synthase 2	0.819
**ENSP00000275493**	*mENA*	1956	epidermal growth factor receptor	0.800
**ENSP00000276431**	*DR5*	8795	TNF receptor superfamily member 10b	0.800
**ENSP00000350941**	*SRC*	6714	SRC proto-oncogene, non-receptor tyrosine kinase	0.800
**ENSP00000354612**	*PTGS1*	5742	prostaglandin-endoperoxide synthase 1	0.800
**ENSP00000370989**	*CD274*	29126	CD274 molecule	0.800
**ENSP00000373691**	*DUOX2*	50506	dual oxidase 2	0.800
**ENSP00000430684**	*IKBKB*	3551	inhibitor of kappa light polypeptide gene enhancer in B-cells, kinase beta	0.800
**ENSP00000303706**	*CDC25A*	993	cell division cycle 25A	0.800
**LACT**				
**Protein**	**Gene Symbol**	**Gene ID**	**Gene Name**	**Score**
**ENSP00000231751**	*LF*	4057	lactotransferrin	0.177
**ENSP00000261024**	*SLC40A1*	30061	solute carrier family 40 member 1	0.156
**ENSP00000327758**	*CSX1*	1482	NK2 homeobox 5	0.220
**ENSP00000336764**	*OPRL1*	4987	opioid related nociceptin receptor 1	0.228

**Table 2:** Enrichment analysis results for MES and LACT interactions using Gene Ontology tools.

ID	Description	Gene Ratio	Adj. *p* MES	Gene Ratio	Adj. *p* LACT	Adj. *p* Joint
**GO:0007193**	adenylate cyclase-inhibiting G-protein coupled receptor signaling pathway	8/169	6.48 × 10^−7^	1/4	0.01589	1.03 × 10^−8^
**GO:0045071**	negative regulation of viral genome replication	5/169	0.00042	1/4	0.01398	5.88 × 10^−6^
**GO:0043066**	negative regulation of apoptotic process	11/169	0.01080	3/4	0.00519	0.00006
**GO:0051092**	positive regulation of NF-κB transcription factor activity	7/169	0.00207	1/4	0.03191	0.00007
**GO:0045454**	cell redox homeostasis	4/169	0.01428	1/4	0.02089	0.00030
**GO:0043123**	positive regulation of I-κB kinase/NF-κB signaling	6/169	0.01156	1/4	0.03764	0.00044
**GO:0006959**	humoral immune response	3/169	0.02928	1/4	0.01759	0.00052
**GO:0045669**	positive regulation of osteoblast differentiation	3/169	0.03114	1/4	0.01760	0.00055
**GO:0045944**	positive regulation of transcription from RNA polymerase II promoter	16/169	0.04039	2/4	0.02089	0.00084
**GO:0005506**	iron ion binding	5/168	0.01795	1/4	0.04900	0.00088
**GO:0001503**	ossification	3/169	0.04643	1/4	0.02089	0.00097
**GO:0090575**	RNA polymerase II transcription factor complex	2/175	0.04601	1/4	0.02134	0.00098
**GO:0001934**	positive regulation of protein phosphorylation	4/169	0.04414	1/4	0.03058	0.00135
**GO:0016323**	basolateral plasma membrane	5/175	0.03440	1/4	0.04018	0.00138

Adj. *p*—adjusted *p*-value; GO—Gene Ontology; ID—identifier; LACT—lactoferrin; MES—mesalazine; NF-κB—nuclear factor kappa-light-chain-enhancer of activated B cells.

**Table 3:** Binding affinities of MES with CCND1, MYC, and lactoferrin.

Protein	ΔG [kcal/mol]
LACT	−6.7
CCND1	−5.4
MYC	−5.0

CCND1—cyclin D; MYC—myelocytomatosis oncogene; MES—mesalazine.

## Data Availability

Processed data are contained within the article. Raw data are available on request.
